# Carotid near-occlusion is often overlooked when CT angiography is assessed in routine practice

**DOI:** 10.1007/s00330-019-06636-4

**Published:** 2020-01-31

**Authors:** Elias Johansson, Thomas Gu, Richard I. Aviv, Allan J. Fox

**Affiliations:** 1 Department of Clinical Science, Umeå University, Umeå, Sweden; 2 Wallenberg Center for Molecular Medicine (WCMM), Umeå University, Umeå, Sweden; 3 Department of Radiology, Division of Neuroradiology, University of Ottawa, Ottawa, Canada; 4 Department of Medical Imaging, Sunnybrook Health Science Center, University of Toronto, Toronto, Canada

**Keywords:** Carotid stenosis, Computed tomography angiography, Stroke

## Abstract

**Objective:**

Assess the sensitivity and specificity of computed tomography angiography (CTA) for carotid near-occlusion diagnosis interpreted in clinical practice against expert assessment.

**Methods:**

CTAs were graded by two expert interpreters for near-occlusion. Findings were compared with clinical reports in 383 consecutive cases with symptomatic ≥ 50% carotid stenosis. In addition, 14 selected CTA exams (8 near-occlusions and 6 controls) were analyzed in a national effort by 13 radiologists experienced with carotid CTA.

**Results:**

In clinical practice, imaging reports were 20% (95% CI 12–28%) sensitive for near-occlusion, ranging 0–58% between different radiologists; specificity was 99%. Among the 13 radiologists reviewing the same 8 near-occlusions, the average sensitivity was 8%, ranging 0–75%; specificity was 100%.

**Conclusions:**

Carotid near-occlusion is systematically under-reported in clinical routine practice, caused by limited application of grading criteria when assessing CTA.

**Key Points:**

• *Carotid near-occlusion is severe stenosis with distal artery collapse; this collapse is often subtle.*

• *A fifth of near-occlusions were detected in routine practice. Many readers mistake near-occlusion for stenosis without distal artery collapse, either by not actively searching for subtle collapses or by not interpreting the collapse correctly when noticed.*

• *On the other hand, the novice diagnostician should be cautioned to not over-diagnose near-occlusion; other causes of extracranial ICA asymmetry also exist such as distal disease and Circle of Willis anatomical variants.*

## Introduction

**Fig. 1:**
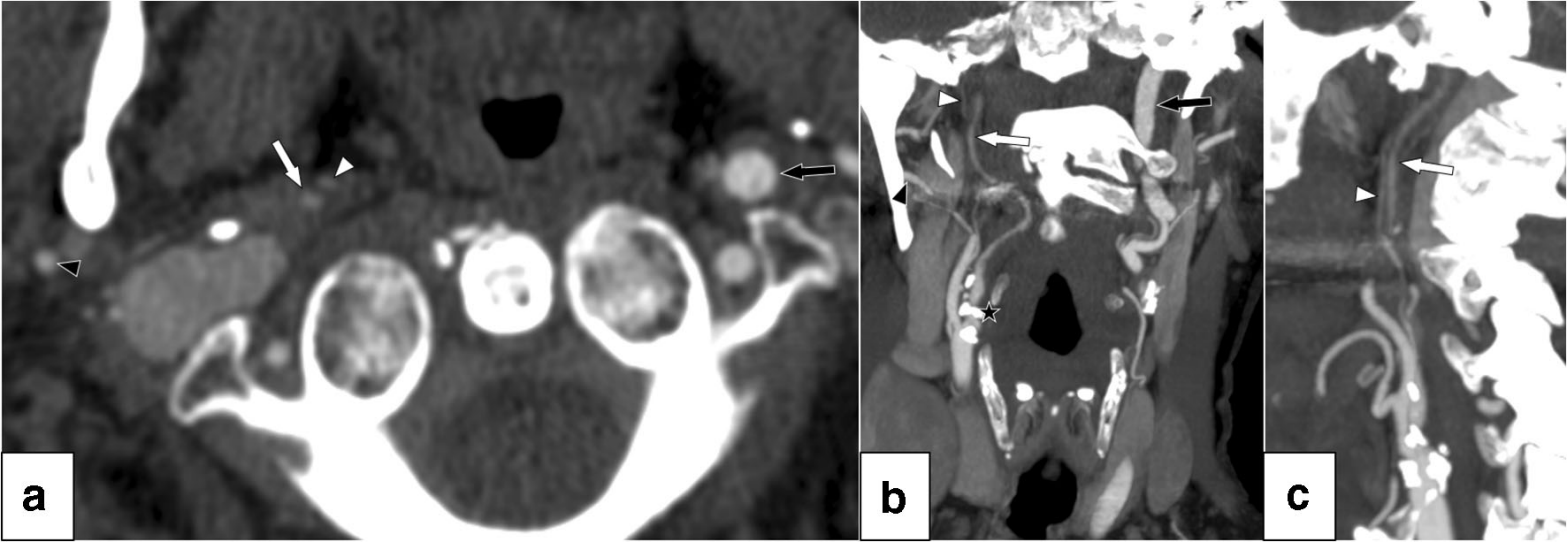
Near-occlusion with full collapse. **a** Axial view of distal findings at C1/C2 vertebrae level. Right distal ICA is tiny (white arrow), not much larger than ascending pharyngeal artery (white arrowhead) but larger than ipsilateral ECA (black arrowhead) and contralateral ICA (black arrow). **b** Coronal view. Stenosis hard to visualize due to severe calcifications (black star). **c** Sagittal view of distal ICA (proximal ICA not in plane). This case was correctly identified as near-occlusion in routine practice and by 2 of the 13 Swedish radiologists

**Fig. 2:**
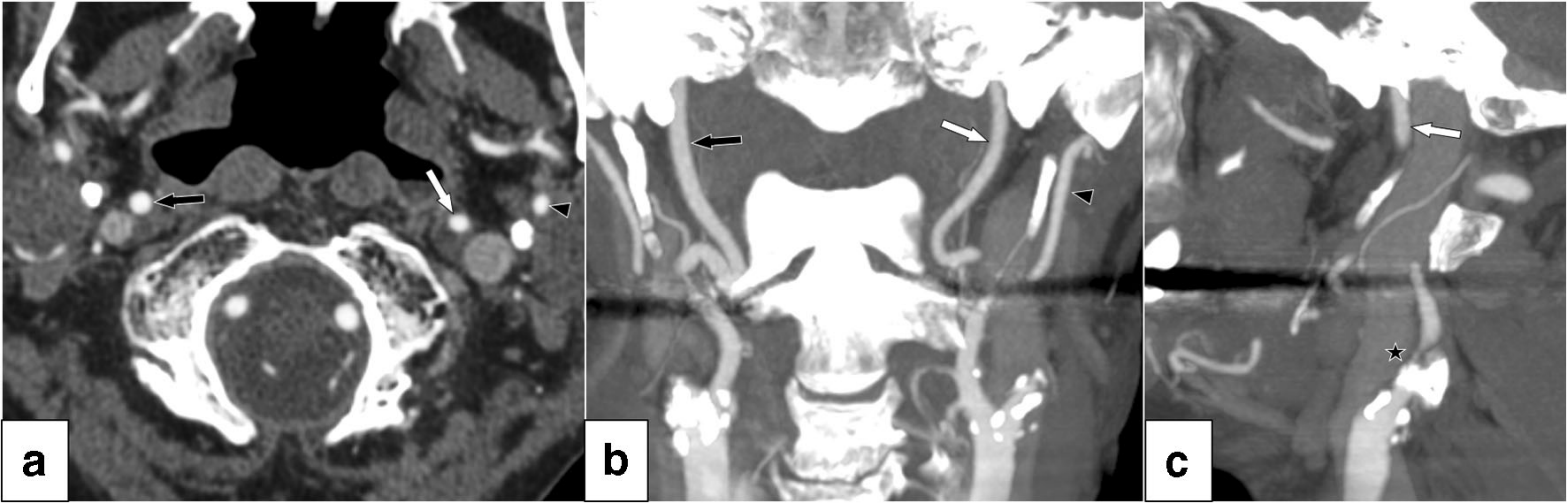
Near-occlusion without full collapse. **a** Axial view of distal findings at C1 vertebra level. The left distal ICA (white arrow, diameter 2.8 mm) is small, smaller than right ICA (black arrow, diameter 3.9 mm) and similar to left ECA (black arrowhead, 2.7 mm). **b** Coronal view, clearly demonstrating ICA asymmetry and normal-appearing, albeit small, distal ICA. Proximal ICA including good view of stenosis not in plane. **c** Sagittal view. Stenosis partly visible, partly obscured by calcifications (black star). Most distal part of ICA visible, but most of ICA not in plane. This case was interpreted as a conventional stenosis in routine practice and by 12 of the 13 of Swedish radiologists

Carotid near-occlusion is a variant of severe carotid stenosis. In contrast to conventional ≥ 50% carotid stenosis, near-occlusion has a reduced artery size (“collapse”) distal to the stenosis [1–3]. The collapse is a physiological response to reduction in flow. When the flow reduction is severe, the distal artery has a threadlike appearance (near-occlusion with full collapse) (Fig. 1); with less severe flow reduction, the distal artery shows a “normal-appearing” but small distal artery (near-occlusion without full collapse) [1–3] (Fig. 2). Near-occlusions need to be distinguished from conventional ≥ 50% stenosis because guidelines recommend medical therapy alone for symptomatic near-occlusion but revascularization for conventional ≥ 50% stenosis [4, 5]. Because near-occlusions show partially collapsed internal carotid arteries (ICA), NASCET prescribed that percent carotid stenosis not be calculated in cases of near-occlusion as this would produce fallacious stenosis assessments [1–3, 6].

Near-occlusion can be shown by computed tomography angiography (CTA) [1, 2, 7, 8]. However, literature review suggests that many clinicians remain unaware of the existence of near-occlusion without full collapse as a lesser near-occlusion, thus recognizing only near-occlusion with full collapse [2]. A review of several cerebral angiography textbooks yielded no reference to near-occlusions with a “normal-appearing” albeit small distal artery [9–13]. Similarly, no description drawing attention to the near-occlusion diagnosis as including more than just full near-occlusion was found [9–13]. Hence, it is plausible that incomplete knowledge, lack of attention to detail, and inaccurate interpretation or synthesis of CTA findings might cause many near-occlusions to be missed for diagnosis in routine practice. A reasonable way to assess this is to compare routine practice grading of CTA with expert grading, but no study has performed such a comparison.

The purpose of this study was to assess the sensitivity and specificity of CTA for carotid near-occlusion diagnosis, interpreted in routine clinical practice compared with expert assessments.

## Methods

**Fig. 3:**
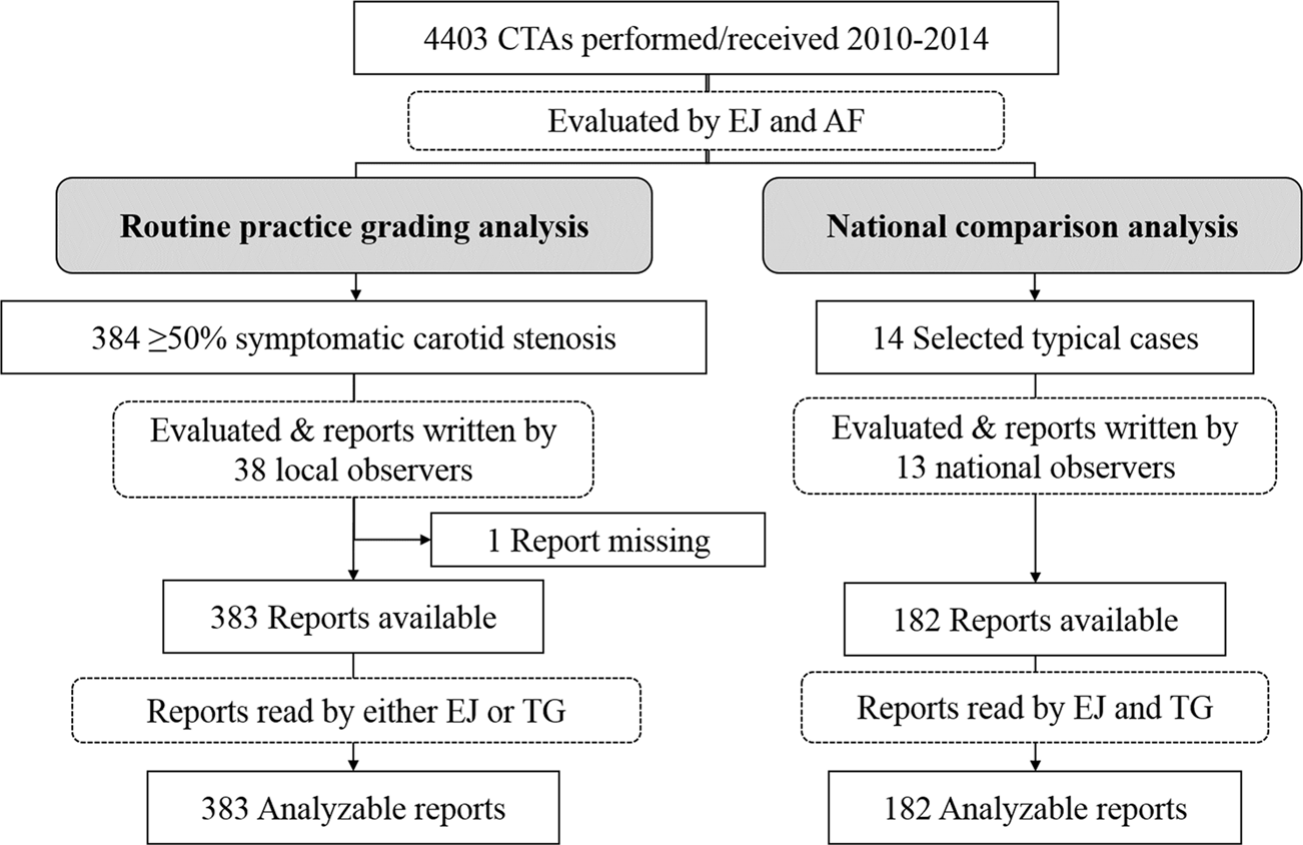
Study flow chart

We evaluated a consecutive sample of CTAs from all patients aged ≥ 18 years performed at or transferred to the Radiology Department at the University Hospital of Northern Sweden, Umeå, Sweden, between 2010 and 2014 (Fig. 3). CTAs were performed using several machines and protocols locally and at all referring sites, none adding delayed phase imaging. All 4403 exams (4042 patients) were re-evaluated by one observer (E.J.) and all cases of suspected near-occlusion or near-occlusion mimics were re-evaluated in a blinded fashion by a second observer (A.F.) with disagreements settled by consensus discussion. All evaluations were performed blinded to original reports; none of which was reported then by E.J. or A.F.. Conventional angiography was rarely used in the clinical setting; only CTAs were assessed in this study. The study was approved by the ethical review board in Umeå with need for informed consent waived due to the observational nature of the study.

### CTA image interpretation

Near-occlusion was diagnosed when severe carotid stenosis was shown with a reduced distal internal carotid, compared with expected. Near-occlusions with full collapse showed a threadlike ICA distally, clearly smaller than the ECA (exception: where ECA was also size-reduced). Near-occlusions without full collapse had a normal-appearing albeit relatively small distal ICA. In the main analysis, a case of near-occlusion was determined in accordance with the approach of previous studies [3, 8, 14], i.e., systematic interpretation of several features: Luminal reduction had to be visible as extracranial ICA asymmetry [8], or clearly size-reduced when compared with external carotid artery (ECA) in cases of bilateral near-occlusion or contralateral occlusions. An interpretive approach was used, weighing the information of the four measurable features of near-occlusion (stenosis severity, distal ICA size, distal ICA asymmetry, and ICA/ECA ratio) to find the most appropriate rating(s) for the findings [8, 14]. A conservative approach was used, reserving the diagnosis of near-occlusion only when it was the most reasonable diagnosis.

An important mimic that was accounted for was anatomical variance for distal ICA size associated with variations of the Circle of Willis [7, 14]. The opposite ICA, usually supplying the middle cerebral artery and an anterior cerebral artery, can be larger when supplying a fetal origin posterior cerebral artery and/or both anterior cerebral arteries. Conversely, the ICA can be smaller when not contributing to the anterior or posterior cerebral artery. When the smaller ICA of such asymmetry coincides with a stenosis, it can mimic near-occlusion [7, 14]. Cases that turned out to be a near-occlusion mimic by full assessment were categorized as conventional stenosis.

Occlusion was diagnosed when no contrast was seen beyond the stenotic lesion, commonly with a rounded stump of contrast filling. Cases with collapsed contrast visible distal to the stenosis but had not yet reached skull base at time of image capture were diagnosed as near-occlusion with full collapse. This was on the basis that, to be filled at the moment of the early bolus slicing on CTA, contrast requires a distal outlet that may not be visible without delayed imaging. Among cases with conventional stenosis, degree of stenosis was measured using established NASCET criteria [6].

As an additional explorative method, near-occlusion can be suggested when it fulfills 4 criteria proposed by Bartlett et al [8], i.e., stenosis diameter ≤ 1.3 mm, distal diameter ≤ 3.5 mm, ICA asymmetry with a ratio of ≤ 0.87, and ICA/ECA ratio ≤ 1.27, excluding cases with stenosis too calcified to assess diameter and/or contralateral ICA occlusion.

Measurements were made by the observer (E.J.) utilizing NASCET methodology. Distal ICA was assessed and measured well beyond the bulb where the walls are parallel and ECA was assessed and measured just proximal to its terminal bifurcation behind the jaw, both usually in the same axial slice at C2 vertebral level (Fig. 2). Calipers were systematically placed in the middle of the “fuzzy edge,” without extra magnification making the edge appear wider. When feasible, measurements were taken from axial source images, starting with standard windows (e.g., 700 HU width, centered at 200 HU) and thereafter freely adjusted to optimally show lumen. Appropriate window settings have multiple variables, depending on scan timing and cardiac physiology, as well as atherosclerotic disease, so that windowing needs customization for each artery reflecting clinical practice. Axial source images were preferred, but coronal and sagittal maximum intensity projections (MIP) and multiplane reconstructions (MPR) were also interpreted. In cases of very severe stenosis, where partial volume effects make tiny lumen hard to visualize, stenosis diameter was arbitrarily set to 0.5 mm.

### Local CTA comparison

Expert diagnoses were compared to original imaging reports. We included all patients with symptomatic ≥ 50% carotid stenosis with CTA performed for preoperative evaluation. In 5 patients, there were two instances of having a symptomatic stenosis—on the contralateral side and/or > 1 year since last ipsilateral event; the second instance was also included. Imaging reports of diagnoses as near-occlusions on expert grading were originally written by nine neuroradiologists (eight experienced, one recently completed training) and 29 general radiologists. The symptomatic side was analyzed. In order to categorize the report findings, the reports were assessed by two stroke clinicians with near-occlusion expertise (E.J., T.G.), blinded to the grade of stenosis but aware of symptomatic side. Any synonym or approximating descriptors of near-occlusion was considered but general expressions like “very severe stenosis” were not as they refer to the stenosis, not the distal ICA. Reports were categorized as near-occlusion when a near-occlusion term was used to clearly or possibly describe a severe stenosis associated with a small distal artery and near-occlusion was the only diagnosis. Reports with near-occlusion terms was clearly used to describing the severe stenosis, not weighing in a small distal artery, was not considered near-occlusion, even when small distal artery was also mentioned in passing. In addition, clinical records were assessed to understand what degree of stenosis the clinician used for the treatment decision.

### National CTA comparison

As an additional comparison, 14 selected cases were sent to other institutions: The radiology departments of all 52 Swedish hospitals managing acute stroke were contacted by telephone. From this, 13 radiologists from 11 hospitals agreed to participate in this study. Academic centers were overrepresented (43% participation) compared with non-academic centers (18% participation). All participating radiologists graded carotid stenoses with CTA as their routine; 9 estimated that they had evaluated > 100 CTA carotid exams in their lifetime. All five participants from academic centers were neuroradiologists; no neuroradiologist worked outside. None of the participants had any previous professional contact with this study group. Care was taken to not divulge the purpose of the study to the participants other than being told the study was assessing how carotid stenosis is graded in routine practice. Study results and purpose were presented to the participants after study completion with options to withdraw participation; however, none opted to do so and none claimed misinterpretation of their findings after feed-back.

We selected 14 cases, sent to participating radiologists for interpretation. The selected cases were 4 conventional ≥ 50% stenosis, 5 near-occlusion without full collapse, 3 near-occlusions with full collapse, and 2 occlusions. All chosen exams had perfect inter- and intra-rater agreement and were judged as typical examples by both E.J. and A.F.. In six of the near-occlusions, the four features of near-occlusion were easily assessable and two were more difficult: One near-occlusion without full collapse had a very calcified stenosis and one near-occlusion with full collapse had a very small residual distal lumen, but visible from bulb to terminus. All eight near-occlusion cases fulfilled all four measured Bartlett criteria except the case with very calcified stenosis where stenosis diameter could not be determined.

We sent axial source images and 3 mm MIP reformats in axial, coronal, and sagittal planes. Participants were instructed to treat these as routine cases (free to use any machine setting, reformats, etc.) and provide an imaging report. The imaging reports were categorized as the original reports in clinical routine practice by two observers (E.J. and T.G.) blinded to case details and to each other. Disagreements between observers were resolved by consensus discussion.

### Analysis and statistics

Stenosis grading from the CTA expert review was the reference standard. CTA imaging reports (local and national comparisons) were compared with the reference standard using mean, standard deviation (SD), 95% confidence intervals (95% CI), 2-sided *χ*^2^ test, and one-way ANOVA with REGW-Q post hoc test. For reliability testing, kappa values were used. *P* < 0.05 was pre-specified as border for statistical significance. SPSS 24.0 was used in all calculations.

## Results

In the routine practice grading analysis, 384 CTA exams from 379 consecutive patients were included, but 1 image report was missing, resulting in 383 exams with data available (baseline data presented in Table 1). There were three bilateral near-occlusions, with ICAs ranging 0.8–2.8 mm and clearly smaller than the ECA bilaterally.

**Table 1:** Baseline characteristics

	Included exams (*n* = 383)
Age mean (SD)	71.8 (8.3)
Men, *n* (%)	265 (69)
Previous stroke, *n* (%)	56 (15)
Previous myocardial infarction, *n* (%)	70 (18)
Current angina, *n* (%)	55 (14)
Current smoker, *n* (%)	68 (18)
Diabetes, *n* (%)	94 (25)
Hypertension*, *n* (%)	340 (89)
Previous arterial revascularization, *n* (%)	69 (18)
On antiplatelet or anticoagulant treatment when seeking health care, *n* (%)	167 (44)
On lipid-lowering treatment when seeking health care, *n* (%)	180 (47)
Presenting event: stroke, *n* (%)	194 (51)
Presenting event: TIA, *n* (%)	136 (36)
Presenting event: retinal^†^, *n* (%)	53 (14)
Conventional ≥ 50% stenosis, *n* (%)	279 (73)
Near-occlusion without full collapse, *n* (%)	57 (15)
Near-occlusion with full collapse, *n* (%)	47 (12)
Sought health care on the day of presenting event, *n* (%)	290 (76)
Days between presenting event and CTA exam median (IQR)	3 (0–7)
Undergoes carotid revascularization, *n* (%)	223 (58)

*IQR*, inter-quartile range; *SD*, standard deviation; *TIA*, transient ischemic attack

*First recorded blood pressure ≥ 140 systolic, ≥ 90 diastolic, and/or use of blood pressure medication

^†^Amaurosis fugax or retinal artery occlusion

**Fig. 4:**
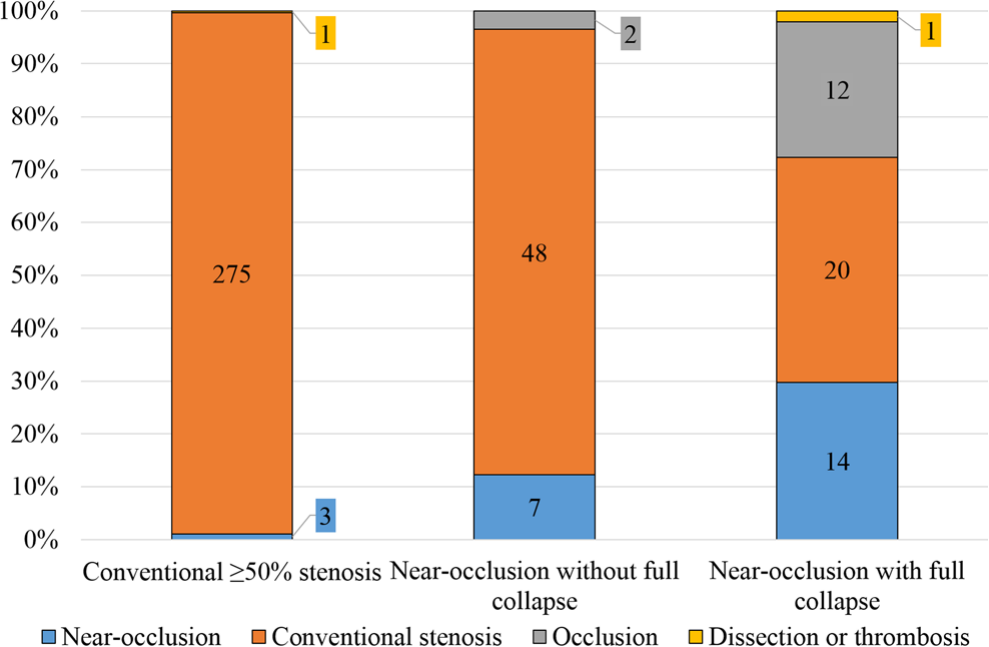
Routine practice grading analysis, comparing expert grading of CTA (3 bars) and imaging reports of the same exam (4 colors)

**Fig. 5:**
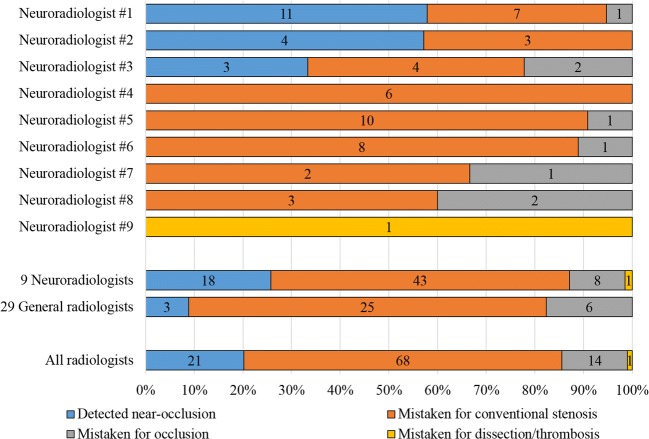
Routine practice grading analysis, comparing expert grading and individual radiologists’ reports. Only near-occlusion cases analyzed. All 9 neuroradiologists are assessed individually; the 29 general radiologist that wrote 1–3 reports each are assessed as a group

Sensitivity of near-occlusion (including those with and without full collapsed) in the original report was 20% (95% CI 12–28%). Specificity of near-occlusion was 99% (Fig. 4). Detected near-occlusions had more prominent ICA asymmetry than near-occlusions mistaken for conventional stenosis; near-occlusions with full collapse were more often detected than near-occlusion without full collapse (Table 2). The vast majority (81%) of missed near-occlusions were called conventional stenosis. Near-occlusion sensitivity ranged 0–58% among the nine participating neuroradiologists (Fig. 5). Two neuroradiologists with > 50% sensitivity were the most experienced, one recently completed training. Among 29 general radiologists who reviewed 1–3 exams with near-occlusion (34 exams in total), three correctly diagnosed a single near-occlusion (Fig. 5).

**Table 2:** Image features of near-occlusions detected and overlooked in routine practice. Single case of near-occlusion with full collapse mistaken for dissection/thrombosis excluded

	Near-occlusions			*p*
	Detected (*n* = 21)	Mistaken for conventional stenosis (*n* = 68)	Mistaken for occlusion (*n* = 14)	
Stenosis diameter mm mean (SD)	0.7 (0.2)	0.7 (0.2)	0.6 (0.4)	0.31*
Distal ICA diameter mm mean (SD)	1.9 (0.9)	2.4 (1.0)	0.8 (1.2)	< 0.001*^,†^
Ipsilateral/contralateral distal ICA mean (SD)	0.48 (0.36)	0.53 (0.21)	0.18 (0.27)	< 0.001*^,†^
Ipsilateral distal ICA/ECA mean (SD)	0.59 (0.30)	0.82 (0.44)	0.50 (1.07)	0.06*
Without full collapse, *n* (%)	7 (12)	48 (84)	2 (4)	< 0.001^#^
With full collapse, *n* (%)	14 (30)	20 (43)	12 (26)	

*ECA*, external carotid artery; *ICA*, internal carotid artery; *SD*, standard deviation

*One-way ANOVA

^†^Post hoc: Only mistaken for occlusion group was separated from the two other groups at *p* < 0.05

^#^2-sided *χ*^2^ test

Of the 104 near-occlusion cases, 21 (20%) were detected, 33 (32%) included no near-occlusion term or mention of distal ICA, and 15 (14%) were mistaken for occlusion; remaining 35 (34%) had some mention or indication of near-occlusion in the report (Table 3). Of 104 local near-occlusion cases, clinicians perceived the degree of stenosis to be near-occlusion in 18% (*n* = 19) and varied with diagnosis in the CTA report, but also by ultrasound findings (Table 3). Chance of clinicians perceiving the near-occlusion diagnosis tended to be higher among the 21 cases with near-occlusion as sole diagnosis (43%) than the 35 cases with mention or indication of near-occlusion (23%; *p* = 0.14, *χ*^2^).

**Table 3:** Report details and chance for near-occlusion to be perceived by the clinician in routine practice

Diagnostic term used	Basis for term use	Problem	*n* (% of all)	Perceived (%)
Near-occlusion or similar	Small distal ICA	None	21 (20%)	9 (43%)*
	Small distal ICA, also percent diagnosis	Mutually exclusive diagnoses	2 (2%)	1 (50%)
	Severe stenosis. Small distal ICA also mentioned	Accidentally correct terminology and incorrect synthesis of information	7 (7%)^†^	3 (43%)
	Severe stenosis. Small distal ICA not mentioned	Accidentally correct terminology and missed small distal ICA or failed to mention small distal ICA	2 (2%)^†^	0 (0%)
	Unclear	Too short report for data extraction	1 (1%)	0 (0%)
Conventional stenosis or similar	Small distal ICA associated with stenosis, but not as a separate diagnosis	Incorrect terminology	7 (7%)	0 (0%)
	Small distal ICA mentioned but not associated with stenosis	Incorrect synthesis of information or incorrect terminology	16 (15%)	4 (25%)^‡^
	Small distal ICA not mentioned	Missed small distal ICA or failed to mention small distal ICA	33 (32%)	2 (6%)^‡^
Occlusion	Contrast not seen in or beyond stenosis	Missed faint distal contrast	6 (6%)	0 (0%)
	Contrast seen beyond but not in stenosis	Incorrect synthesis of information	8 (8%)	0 (0%)
Thrombosis	Appearance	Missed stenosis as cause	1 (1%)	0 (0%)
All cases			104 (100%)	19 (18%)

*ICA*, internal carotid artery

*Nine of 12 (75%) near-occlusion missed despite being sole and correctly based diagnosis on CTA was affected by a conventional stenosis diagnosis on ultrasound

^†^In these nine cases, it was clear that stenosis impression, almost occluded, was the cause of using a near-occlusion or similar term, not that the distal artery was small

^‡^Four of six (67%) near-occlusions perceived despite not diagnosed on CTA were affected by near-occlusion diagnosis on ultrasound

**Fig. 6:**
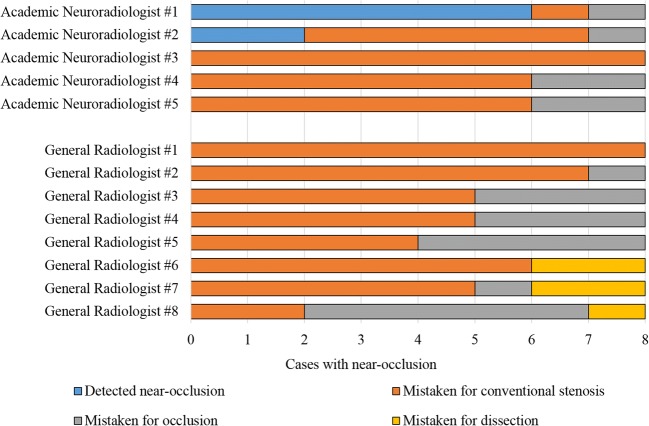
National CTA analysis, comparing expert grading with interpretations of five Swedish Academic radiologists and eight Swedish General Radiologists. The same cases were assessed by all participants; only the eight cases with near-occlusion analyzed

In the national comparison, the 13 participants produced 182 imaging reports; 104 were near-occlusion cases. Near-occlusion sensitivity was 8% (95% CI 2–13%) and specificity was 100% (Fig. 6). Sensitivity ranged from 0 to 75% between observers. Near-occlusion with and without full collapse was similarly often detected (10% and 6%, respectively; *p* = 0.71, *χ*^2^).

Combining the local and national CTA comparison, 16% (8/51) of radiologists that assessed at least one near-occlusion case detected any near-occlusion, higher for neuroradiologists (36%) compared with general radiologists (8%).

Inter-rater reliability between the two expert reviewers for near-occlusion diagnosis on CTA was good: kappa 0.80; intra-rater reliability on 49 randomly selected cases was kappa 0.75 and 0.88 for each observer. The inter-rater reliability between the two readers of CTA reports was good (kappa 0.88, 95% CI 0.79–0.97).

Exploring diagnosing near-occlusion was based on fulfilling 4 of 4 Bartlett criteria, 93% sensitive (87/94) and 97% specific (257/264) compared with expert diagnosis. Of the 94 cases with 4 of 4 Bartlett criteria, local imaging reports diagnosed conventional stenosis or thrombosis in 64 (68%), near-occlusion in 18 (19%), and occlusion in 12 (13%). However, 25 exams (7%, 10 near-occlusions and 15 conventional ≥ 50% stenosis) were excluded from this assessment due to calcified stenosis (*n* = 11), contralateral occlusion (*n* = 12), or both (*n* = 2). All ten false negatives missed a single feature, all well explained by interpretive approach such as bilateral near-occlusion and stenosis proximal to bulb causing ECA collapse. Requiring ≥ 3 Bartlett criteria (including 3 of 3 in cases with a single missing feature) for near-occlusion diagnosis resulted in 100% sensitivity (102/102) and 87% specificity (244/279) compared with expert diagnosis.

## Discussion

The main finding of this study is that near-occlusion is often overlooked when CTA is assessed in routine practice. Near-occlusion sensitivity ranged between 0 and 75% between radiologists, with only 16% of radiologists finding any near-occlusion.

With a paucity of studies reporting grading accuracy of carotid near-occlusion, comparison with similar findings is difficult. Near-occlusion with full collapse is perceived to be the only type of near-occlusion by many [1, 2]. However, since 94% of near-occlusions in the major trial reports were near-occlusions without full collapse [3], it is reasonable to include near-occlusion without full collapse in the definition of near-occlusion. Mistaking near-occlusion without full collapse for conventional stenosis will result in calculating a percent stenosis ratio which is an underestimate of the true stenosis because the distal internal carotid artery lumen is decreased [6]. Given the omission of near-occlusions without full collapse in textbooks [9–13] and much of the literature [1, 2], it is not surprising that radiologists under-recognize it. Indeed, a small distal artery was noted, but not correctly assessed, in many of the near-occlusion cases in this study. Therefore, increasing awareness of near-occlusion without collapse is a global educational priority. Although using 4 of 4 Bartlett criteria was reasonably sensitive (90%) and specific (98%) compared with interpretive approach, the interpretive approach is seemingly superior as causes for not meeting certain thresholds are assessable and cases with missing features can be assessed. Requiring ≥ 3 criteria led to a drop in specificity to 90%. Indeed, superiority of an interpretive approach was concluded in the Bartlett study [8] underscoring the need for an educational effort focused on the interpretive approach, but learners might initially benefit from the Bartlett criteria. A lack of awareness and/or lack of attention to detail leads to symptomatic near-occlusions misdiagnosed as conventional stenosis which, by current guidelines [4, 5], would result in unnecessary treatments. Recent non-randomized evidence has challenged these guidelines [2, 15–22], why further studies and trials might prove superiority of other management strategies. Improved diagnostics may enhance future clinical studies and allow for implementation of findings.

The main analysis of near-occlusion sensitivity was based on original reports finding a 20% sensitivity. This finding was similar (19% sensitivity) when the more easily reproducible 4 of 4 Bartlett criteria approach was used. As each case was assessed by different observers in the original report analysis, it was impossible to separate case and observer dependency. The additional national sample analysis revealed substantial difference in sensitivity (0–75%) despite reviewing the same cases, indicating that variation in sensitivity was caused by difference in observer proficiency. However, near-occlusions with full collapse and smaller distal diameter were more often detected in the original analysis, so the chance of a near-occlusion to be correctly diagnosed was also dependent on case details. Detection of at least one near-occlusion was more common for neuroradiologists than general radiologists, but some neuroradiologists missed all near-occlusions, even those with full collapse. Also, the national sample revealed that the low sensitivity of near-occlusion was not limited to a single center.

Some reports included both a near-occlusion diagnosis and a percentage grade as if they were both correct at the same time. Near-occlusions should not be graded as percent stenosis as deriving percent stenosis is fallacious; these are mutually exclusive [1–3, 22]. We only accepted a near-occlusion term or synonym if it was used as to describe severe stenosis associated with small distal artery, not when describing severe stenosis alone. As near-occlusion is a management altering diagnosis, only correct use of the term should reasonably be acceptable. Many reports mentioned small distal artery; some also associated this with the stenosis. Hence, in these cases, a combination of incorrect terminology and information synthesis caused the missed near-occlusion diagnosis. It is unclear how often the observer saw but did not report a small distal ICA among the cases without mention of small distal ICA; hence, for these cases, it is unclear how often failure to spot the small distal ICA failure to understand the small distal ICA caused the missed near-occlusion diagnosis.

Unfortunately, delayed imaging was not used by all, and conventional angiography was rarely used during the study period (though conventional angiography might not always have a delayed series to show a slowly filling diminutive distal cervical ICA). If used, delayed images might prevent some near-occlusions to be mistaken for occlusions in the original reports. There were possibly cases of near-occlusion only separable from occlusion with delayed imaging, mistaken for occlusion by both the experts and clinical observers. However, interpretation education needs priority as many mistook no visible contrast in severe stenosis lumen and clearly patent arteries beyond the stenosis as occlusion with retrograde filling. With CTA aiming at arterial phase, it is near impossible for contrast to reach from skull base down to bulb region in this short time, rather a technical limitation as partial volume effect is more reasonable to presume. However, the vast majority (81%) of missed near-occlusions were called conventional stenosis—not occlusion.

As the near-occlusion experts conducting the study did not participate in the clinical or diagnostic work during the time of the study, observation bias was reduced. Although we have sampled a consecutive cohort from a single hospital and added scattered observers from a single country, these findings may or may not be representative in other locales, depending on attention to detail of near-occlusions. However, we suspect these results are not specific to Swedish general radiologists or neuroradiologists. As the CTA findings varied with interpreter expertise, CTA findings are likely to vary greatly between centers.

In conclusion, near-occlusion seems to be systematically overlooked in clinical routine practice, presumably due to lack of attention to detail and limited application of documented grading criteria when assessing CTA.

## Abbreviations

CI: Confidence interval
CTA: Computed tomography angiography
ECA: External carotid artery
ICA: Internal carotid artery
MIP: Maximum intensity projections
MPR: Multiplane reconstructions
NASCET: North American Symptomatic Carotid Endarterectomy Trial
SD: Standard deviation
